# Three-Dimensional–guided osteotomy and reconstruction of malunited pediatric capitellum fractures: a report of 2 cases

**DOI:** 10.1016/j.xrrt.2026.100848

**Published:** 2026-08-18

**Authors:** Zisis Terzidis, Eva Brokx, Eline M. van Es, Denise Eygendaal, Christiaan JA. van Bergen

**Affiliations:** Department of Orthopedics and Sports Medicine, Erasmus University Medical Center, Rotterdam, The Netherlands

## Introduction

Isolated capitellum fractures are rare, comprising about 1% of adolescent elbow injuries.^14^ They typically result from a fall on an outstretched hand with the elbow extended,^3^ where force from the radial head fractures the capitellum. These fractures are uncommon under the age of 12 years, due to the capitellum predominantly comprising of cartilage until it is fully ossified around the age of 12 years.^3^ In younger children, the same mechanism is more likely to cause a lateral condylar fracture. A capitellar fracture may be challenging to diagnose due to the immaturity of pediatric bone and the chondral nature of the injury.^3^ Therefore, these injuries are prone to being missed.

**Figure 1:**
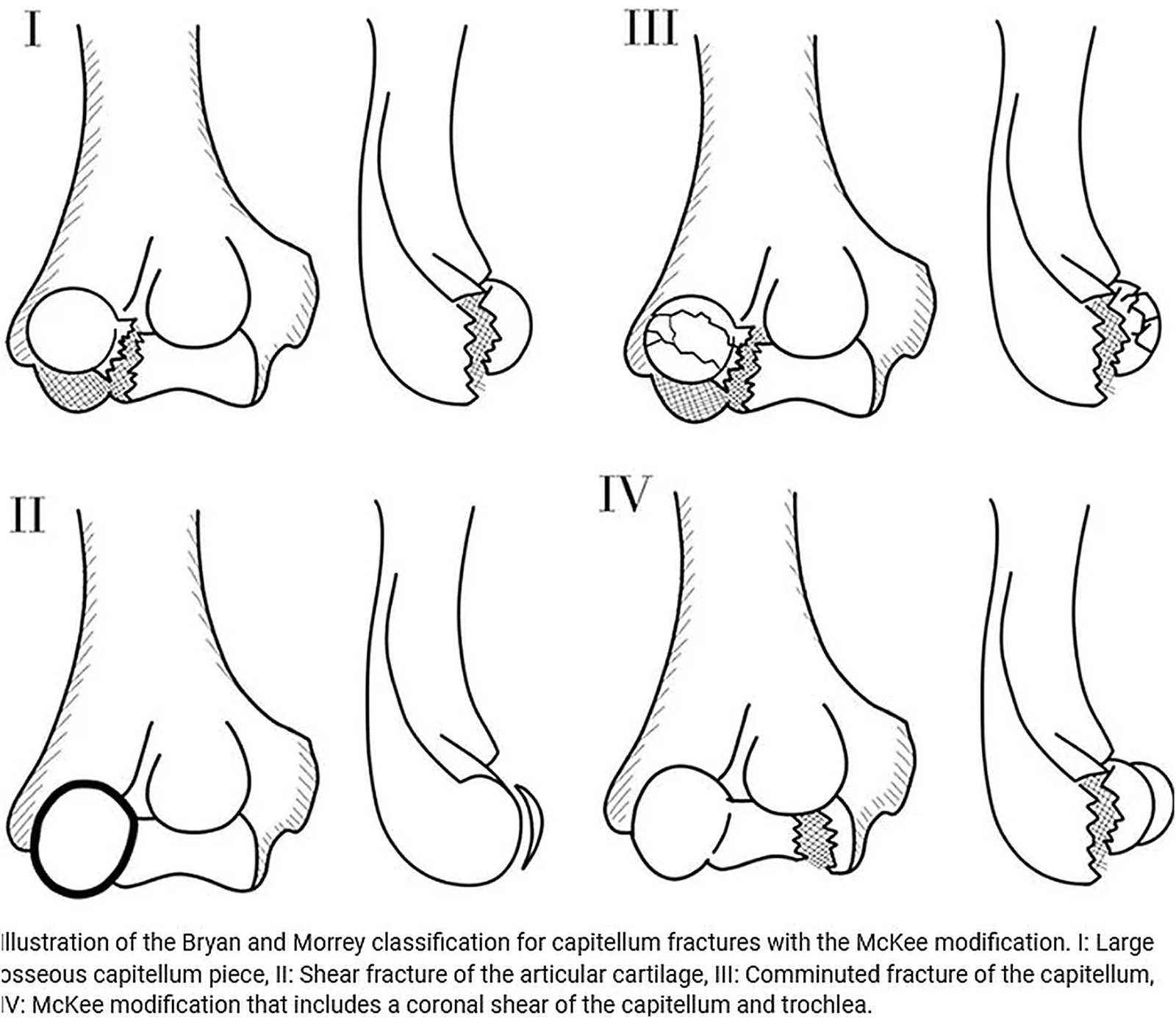
Bryan and Morrey classification.

**Figure 2:**
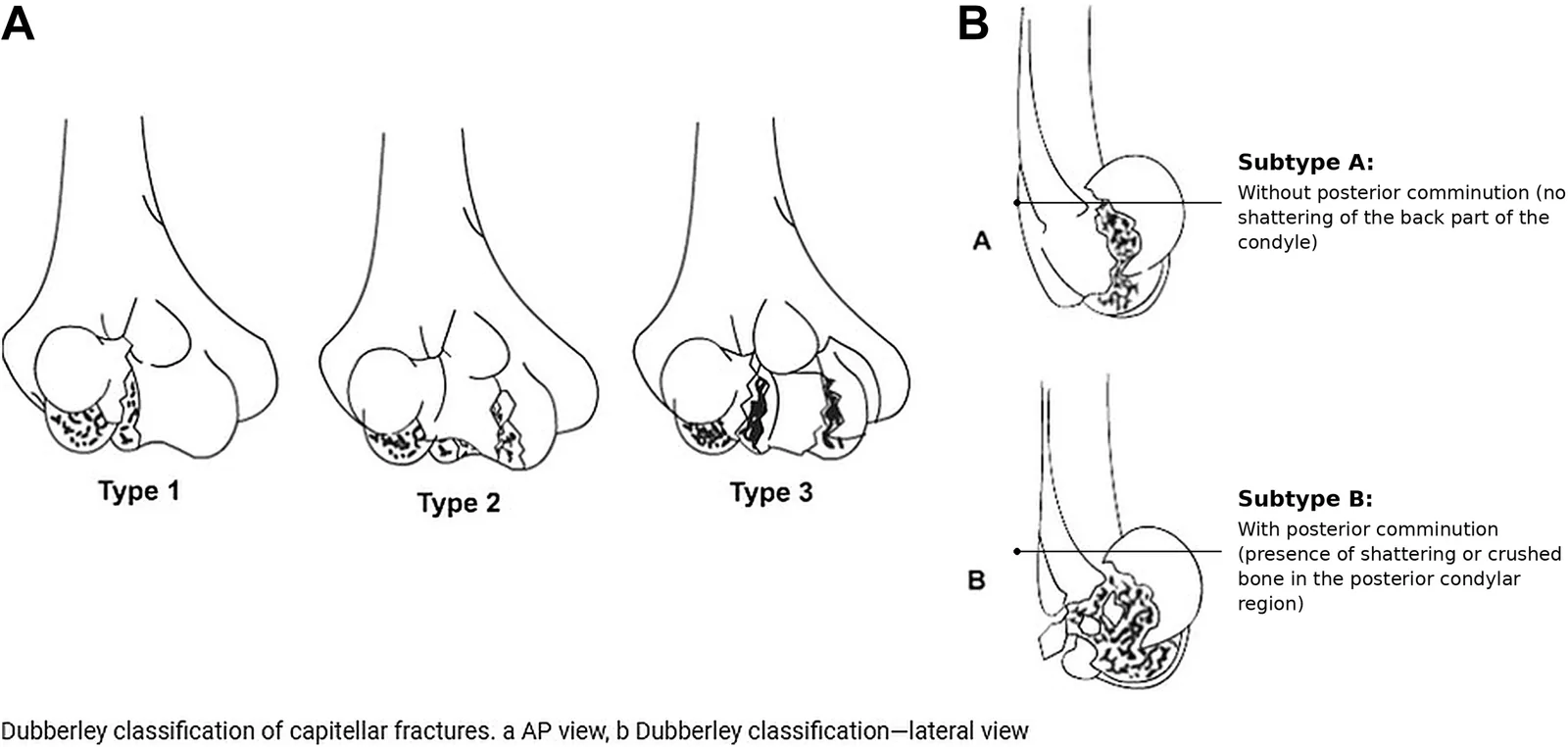
Dubberley classification. (**A**) Without posterior comminution. (**B**) With posterior comminution. *AP*, anteroposterior.

Capitellum fractures are commonly classified using the Bryan and Morrey classification (with the McKee modification) or the Dubberley classification.^1^^,^^16^ Both are displayed in Figures 1 and 2. In the Bryan and Morrey classification, type 1 (Hahn-Steinthal) involves most of the capitellum. Type 2 (Kocher-Lorenz) represents an osteochondral shear fracture with minimal subchondral bone. Type 3 is a comminuted capitellar fracture. McKee later added type 4, describing coronal shear fractures involving both the capitellum and trochlea, often identified by the “doule arc sign” on lateral radiographs.

The Dubberley classification includes type 1 (capitellum ± trochlear ridge), type 2 (single fragment extending into the trochlea), type 3 (separate capitellar and trochlear fragments), and 4 (comminuted fractures). Each type is further subdivided into A (no posterior comminution) and B (with posterior comminution).^1^^,^^13^

Prompt surgical intervention, when indicated, is crucial for optimal outcomes. Delays in treatment can lead to malunion, significant pre-operative discomfort, and a high likelihood of decreased post-operative range of motion.^11^ A prolonged interval between injury and surgery may allow the osseous fragment to heal in an altered position, leading to a malunion of the capitellum. Thus, a delay in treatment may not only cause significant discomfort pre-operatively due to severely restricted range of motion, locking symptoms, and pain, but also likely impact post-operative outcomes. In the event of a malunited capitellum, an osteotomy and reconstruction of the capitellum is needed.

In modern orthopedics, three-dimensional (3D) imaging is used for pre-operative planning, 3D-printed models, and patient-specific instrumentation. 3D imaging has been shown to be helpful in reconstructive surgery of both the lower and upper extremities.^2^^,^^5^^,^^9^ A meta-analysis using 3D imaging for pre-operative planning and 3D printing reported a significant reduction in operative time and intraoperative blood loss during orthopedic trauma surgery.^12^ The use of 3D imaging can greatly aid surgical procedures, particularly for multiplanar deformities, by providing a view of the complex anatomy that cannot be fully captured with conventional two-dimensional imaging. Moreover, 3D imaging enables simulation of corrections, which may lead to more precise surgical strategies. To our knowledge, this technique has not been used for pediatric capitellum malunion.

We present 2 cases of malunited capitellum fractures treated at our tertiary hospital clinic.

## Materials and methods

### Outcome measures

In both cases, information about the patient-related outcome measures (PROMs) was collected at baseline, 6 weeks, 3 months, 6 months, and 1 year. For the first case, longer follow-up data were available. Those PROMs include the Oxford Elbow Score (OES),^7^ Quick Disabilities of the Arm, Shoulder and Hand (QuickDASH),^4^ visual analog scale (VAS) pain, and satisfaction scale. In both cases, informed consent for anonymous use of the data was obtained. This study is a retrospective report of prospectively collected data obtained from the electronic patient records at our institution. The primary objectives of the 3D virtual planning were to restore radiocapitellar joint congruity, correct the elbow axis based on the contralateral template, and regain the original anatomic offset.

The OES consists of the following 3 domains: elbow function, pain, and social-psychological. Each domain contains 4 items, each scored from 0 to 4 (0 representing the greatest severity). A conversion to a metric score yields a value between 0 and 100, with 0 indicating a severe disability and 100 indicating no disability.^7^

The QuickDASH contains 11 questions that assess a patient's ability to perform daily activities and their symptoms, such as pain or discomfort. Unlike the OES, a higher QuickDASH score indicates greater disability, with 0 indicating no disability and 100 representing maximum disability. The QuickDASH has also been validated for pediatric patients.^17^

The visual analog scale (rest-pain and activity-related pain) was collected on a scale from 0 (no pain) to 100 (maximum pain). The satisfaction score ranged from 0 to 10, with 0 indicating not satisfied and 10 indicating super satisfied.

## Results

**Figure 3:**
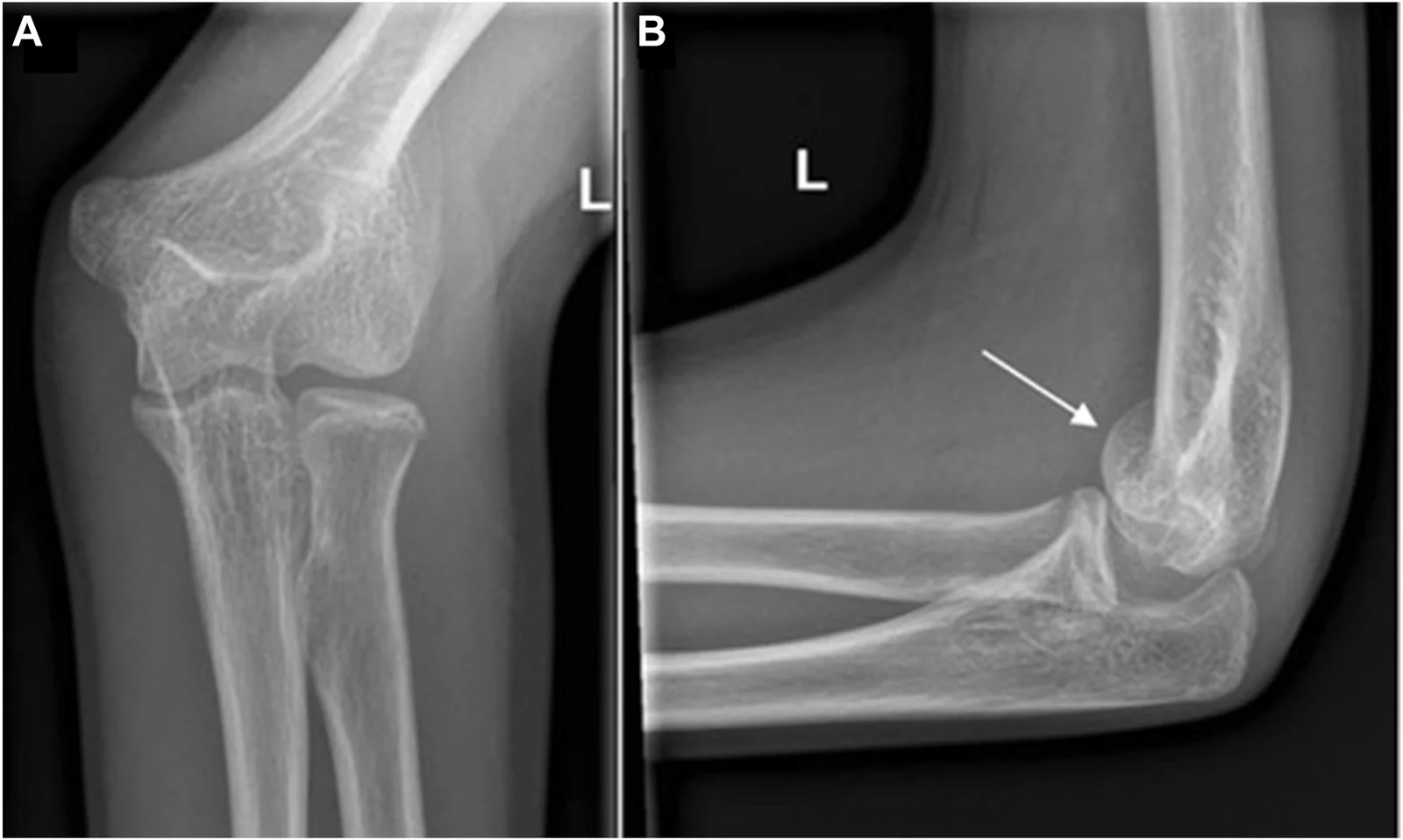
Pre-operative imaging of the elbow joint. (**A**) Anteroposterior (AP) view. (**B**) Lateral view displaying proximal malunion of the capitellum.

The first case concerns a 16-year-old male who was referred to our outpatient clinic with an 8-month-old malunited fracture of the capitellum of the left elbow. The patient sustained the injury due to a biking accident. Due to pain, sourness, and functional impairment, the patient presented to his own general physician 5 weeks after trauma. No injuries were detected on the primary X-rays (Fig. 3, *A* and *B*). Physical therapy was started to treat functional impairment. The impairment persisted, and the patient was referred to a peripheral hospital. A computed tomography (CT) scan of the elbow revealed a malunited capitellum, and the patient was referred to our academic hospital.

The patient presented at our outpatient clinic 8 months after injury with an impairment in flexion and extension of the elbow with alternating joint locking symptoms and a visual analog scale for pain of 6 out of 10. The physical examination showed elbow flexion of 95°, an extension deficit of 60°, pronation of 60°, and supination of 0°; tenderness on palpation of the lateral condyle of the distal humerus and a positive grip-and-grind test was noted, which was performed using axial compression and rotation to identify potential chondral damage or joint incongruity.

**Figure 4:**
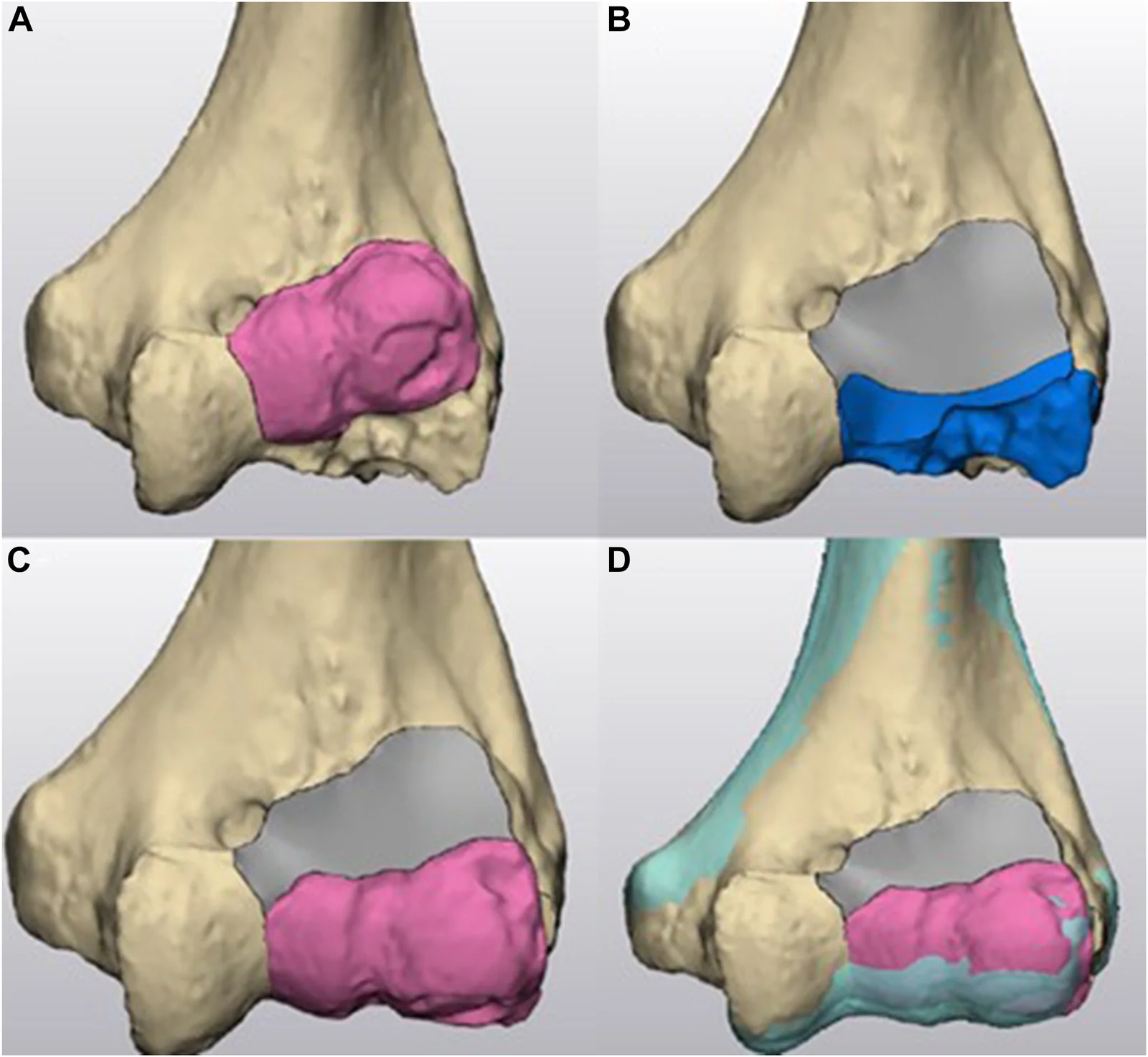
Case 1 - Pre-operative 3D CT reconstruction of the elbow joint. (**A**) Malunited capitellum highlighted in pink. (**B**) Highlighted in blue, the additional bone to be resected before reduction of the capitellar fragment. (**C**) Capitellar fragment after reduction. (**D**) Comparison with contralateral distal humerus (light blue) after reduction. *3D*, three-dimensional; *CT*, computed tomography.

To facilitate precise surgical planning, CT scans of both the ipsilateral and contralateral elbows were obtained. Subsequently, 3D segmentation and virtual planning of the corrective osteotomy were performed, utilizing the contralateral side as an anatomic template to ensure restoration of the native joint geometry (Fig. 4, *A*-*D*, and 5, *A*-*E*). The Kaplan approach was utilized to avoid extensive posterior dissection, thereby preserving the predominant posterior-inferior blood supply to the capitellum and reducing the risk of avascular necrosis, followed by take down of the lateral (ulnar) colateral ligament (LUCL) from its isometric origin. Following the placement of the patient-specific 3D-printed guides, the malunited capitellum was osteotomized and the original site was prepared. The fragment was then anatomically reduced and fixed in the predefined position using 2 cannulated compression screws (Fig. 6, *A* and *B*). Signs of degeneration of the capitellum and radial head were seen. Refixation of the L(U)CL was done with transosseous sutures. Intraoperative function was elbow flexion of 140°, an extension deficit of 10°, pronation of 80°, and supination of 70°. Immobilization with a long arm cast for 2 weeks was followed by static-progressive splinting in extension for 4 weeks. In addition, non-steroid anti-inflammatory drugs were prescribed for three weeks post-operatively to prevent heterotopic ossification.

**Figure 5:**
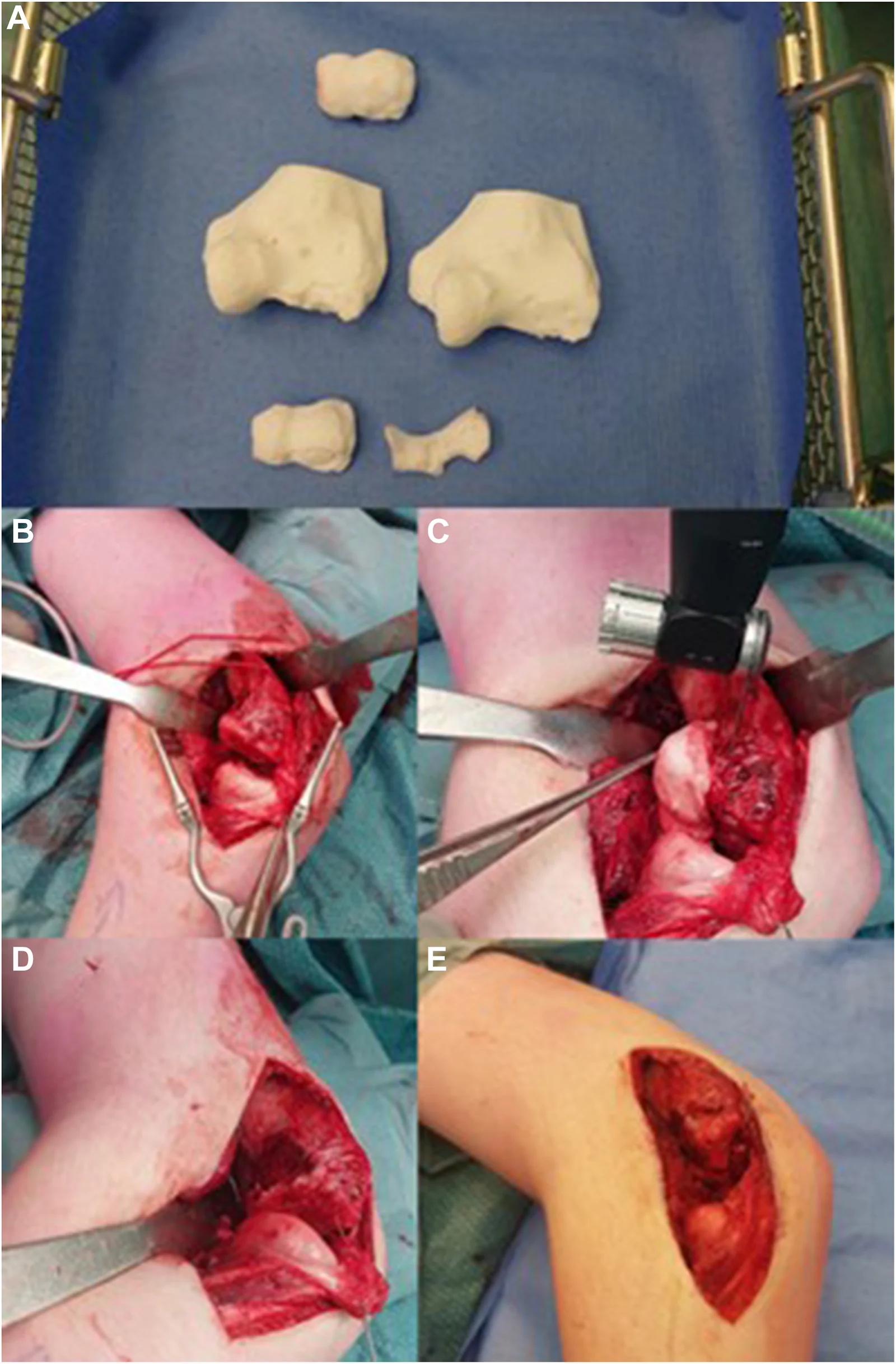
Perioperative imaging demonstrating the reconstruction of the capitellum. (**A**) 3D-printed molds of the malunited capitellum as a guide for the osteotomy. (**B**) Malunited capitellum exposed following the Kaplan approach with LCL takedown. (**C**) Placement of the 3D-printed mold on the capitellum after which osteotomy is performed. (**D**) The elbow joint following the resection of the malunited capitellum fragment (**E**): Reduction of the capitellum fragment in its native location as shown in the 3D reconstruction. Fixation was achieved using 2 cannulated compression screws. *3D*, three-dimensional; *LCL*, lateral collateral ligament.

**Figure 6:**
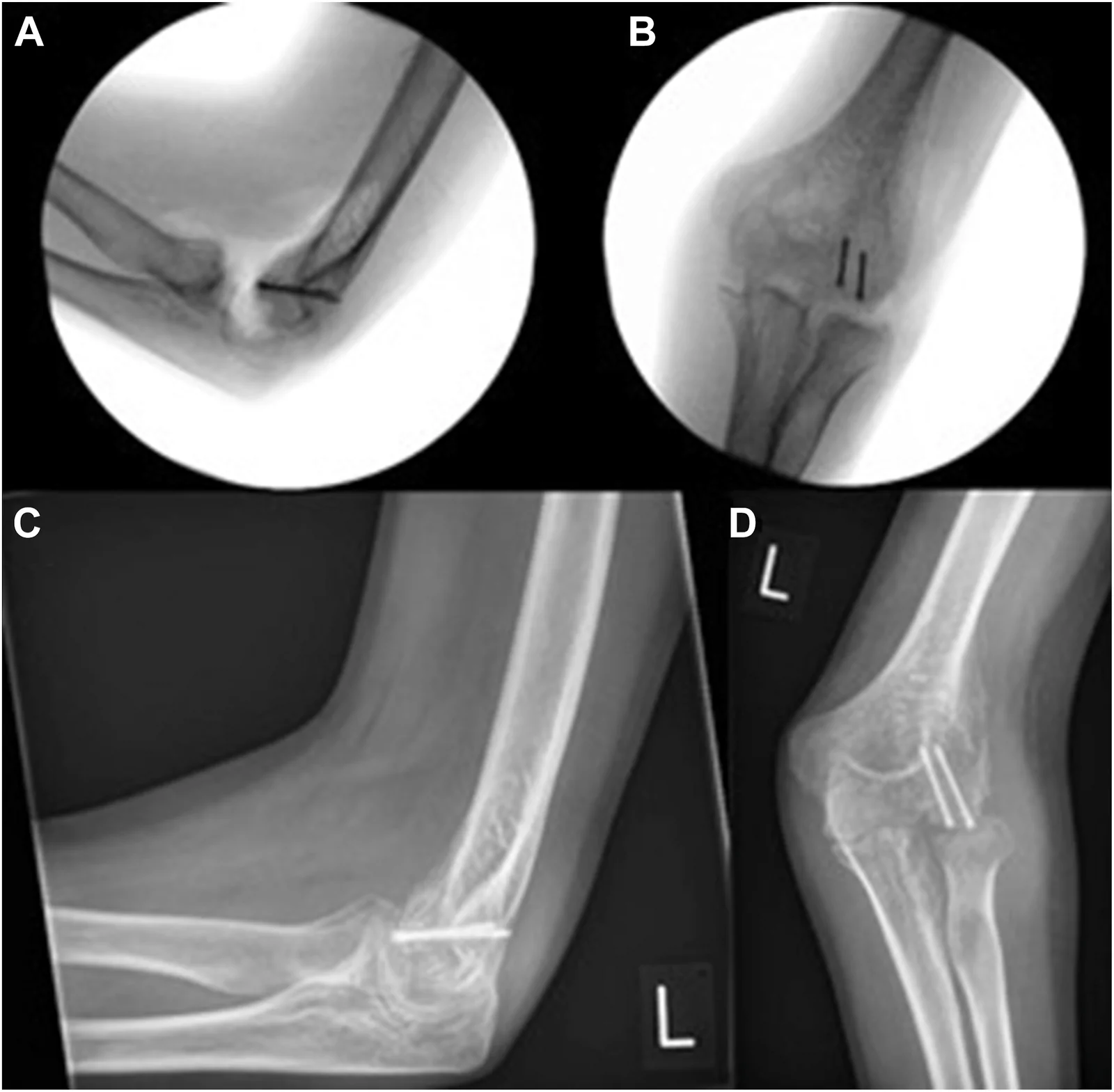
(**A** and **B**) Intraoperative lateral and anteroposterior (AP) imaging of the elbow joint after fixation with cannulated compression screws. (**C** and **D**) Lateral and anteroposterior (AP) imaging at 3-month follow-up.

The 3-month follow-up showed a stiff elbow with elbow flexion of 80°, an extension deficit of 70°, pronation of 30°, and supination of 0°. No complaints of pain were expressed. The X-ray did not show any complications (Fig. 6, *C* and *D*). The static-progressive splinting period was continued with adjusted splints.

At the 6-month follow-up, flexion progressed to 90°, an extension deficit of 60°, and pronation of 80°, with a supination deficit of 10°. Again, no pain complaints were expressed. To assess the causes of elbow stiffness, blood samples were collected and a CT scan was performed. There were no signs of infection. The radiological images showed a healed capitellum osteotomy but with a decreased volume. Patient was admitted for arthrotomy, removal of the screws, culture samples, arthrolysis, and prophylactic release of the ulnar nerve. Surgery was performed 12 months after primary surgery. The intraoperative function was restored from to an elbow flexion of 120° with an extension deficit of 10°. The pronation was restored to 90° and supination to 40°. Post-operatively, the patient started with continuous passive mobilization for 3 days to promote mobility, combined with an non-steroid anti-inflammatory drug and bracing after continuous passive mobilization.

After the second surgery, at the 1-month follow-up patient expressed no pain but some discomfort from the medial side of the elbow to the fourth and fifth digit, classified as radiating ulnaropathy. On physical examination, motor function of the ulnar nerve was intact. The elbow was stiff, with flexion limited to 90°, an extension deficit of 50°, pronation to 70°, and absent supination. Radiographic imaging showed degenerative changes of the elbow joint, involving both the radiocapitellar and ulnohumeral compartments, with enlargement of the radial head and collapse of the capitellum, consistent with the sequelae of avascular necrosis. Physical therapy was continued.

At the 6-month follow-up, the patient reported no pain. Physical examination demonstrated improvement in range of motion, with flexion to 110°, an extension deficit of 30°, pronation to 80°, and supination to 20°. Physical therapy was continued.

At the final follow-up, 1-year post-operatively, the patient remained pain-free. Flexion had further improved to 120°, with a persistent extension deficit of 30°. Radiographic imaging showed no changes compared to prior studies. The patient was subsequently discharged from follow-up.

**Figure 7:**
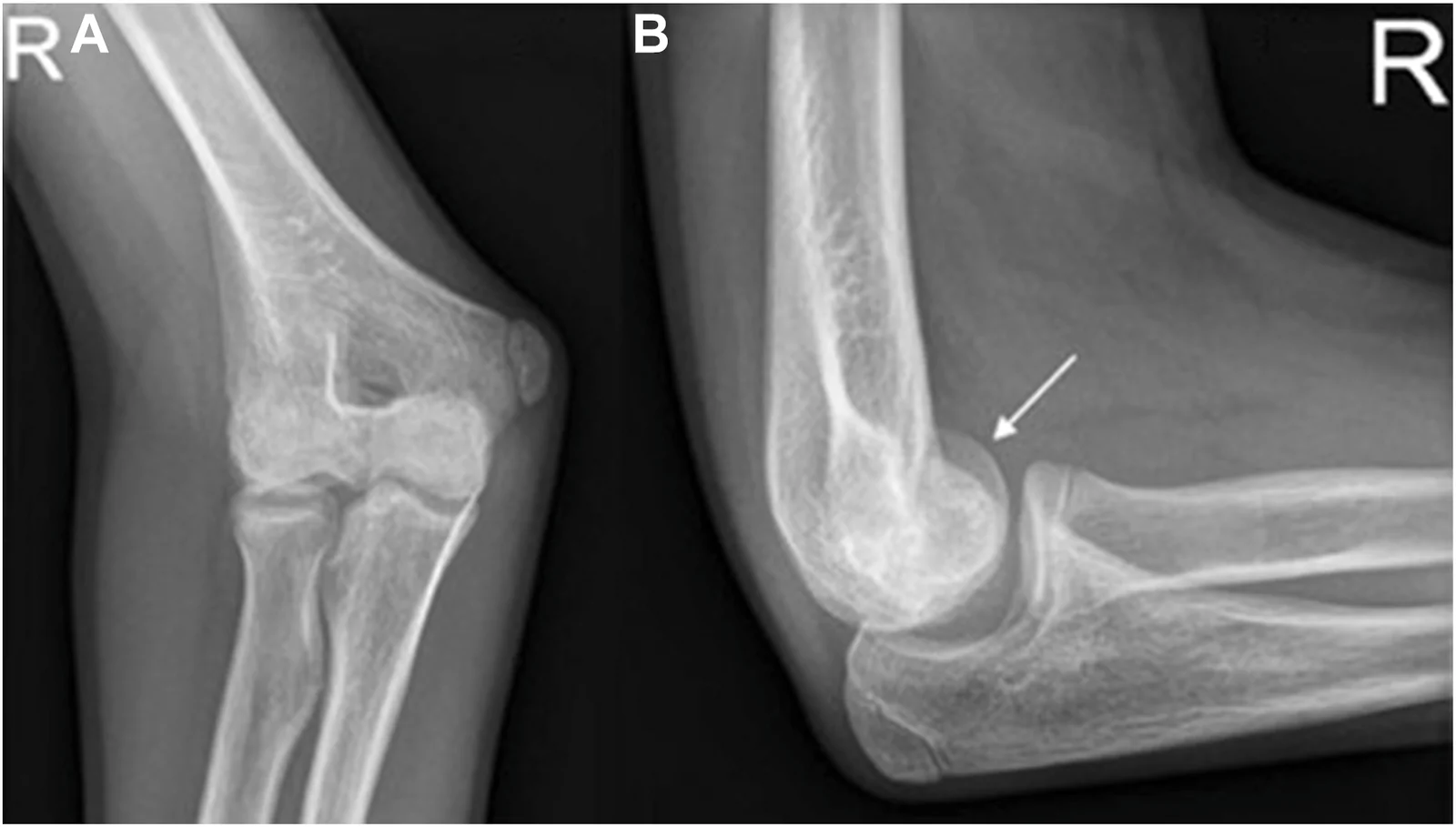
Pre-operative imaging of the elbow joint. (**A**) Anteroposterior (AP) view. (**B**) Lateral view displaying the proximally malunited capitellum fragment.

The second case involves a 13-year-old male referred to our clinic with a malunited right capitellum fracture sustained two months prior from a fall onto a flexed elbow. Due to a persistent impairment in his range of motion, X-rays were obtained five weeks postinjury, which revealed a proximally displaced capitellum fracture (Fig. 7, *A* and *B*). A subsequent CT scan confirmed that the fragment had already reached a state of partial consolidation in its malunited position. At the time of presentation at our outpatient clinic, the patient exhibited a stiff elbow with a restricted range of motion, specifically 120° of flexion and a 60° extension deficit. Forearm rotation was limited to 80° for both pronation and supination. Clinical examination showed tenderness on palpation of the lateral distal humerus, although additional provocative elbow tests, such as the grip and grind test, remained negative.

**Figure 8:**
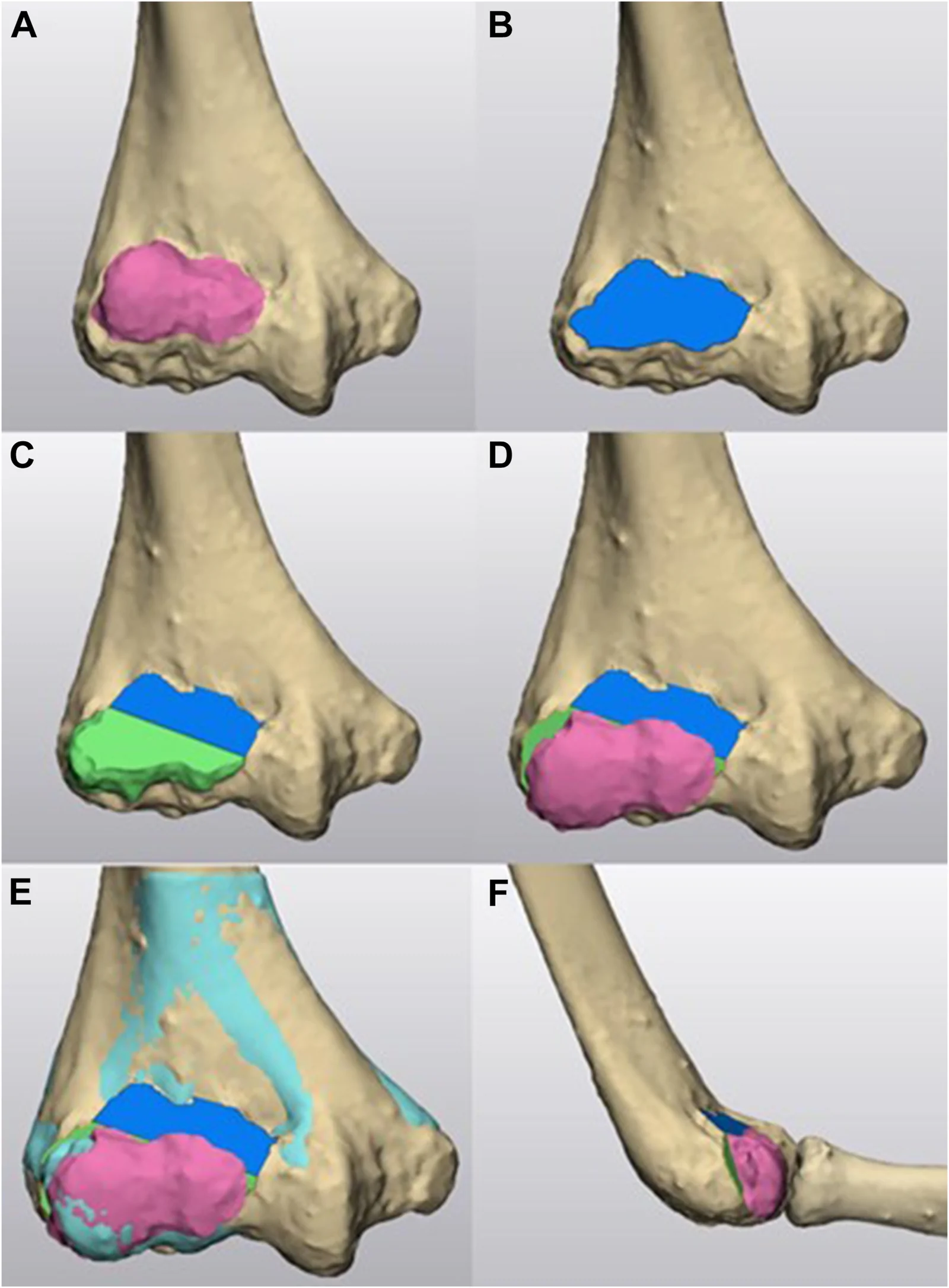
Case 2 - Pre-operative 3D reconstruction of the elbow joint based on CT scans. (**A**) The position of the malunited capitellum. (**B**) Highlighted in blue, the elbow joint after osteotomy of the capitellum. (**C**) In green, the additional bone to be resected before the reduction. (**D**) Reduction of the capitellum according to the 3D reconstruction. (**E**) Comparison with the contralateral distal humerus (light blue) after reduction. (**F**) Post-operative alignment relative to the proximal radius. *3D*, three-dimensional.

**Figure 9:**
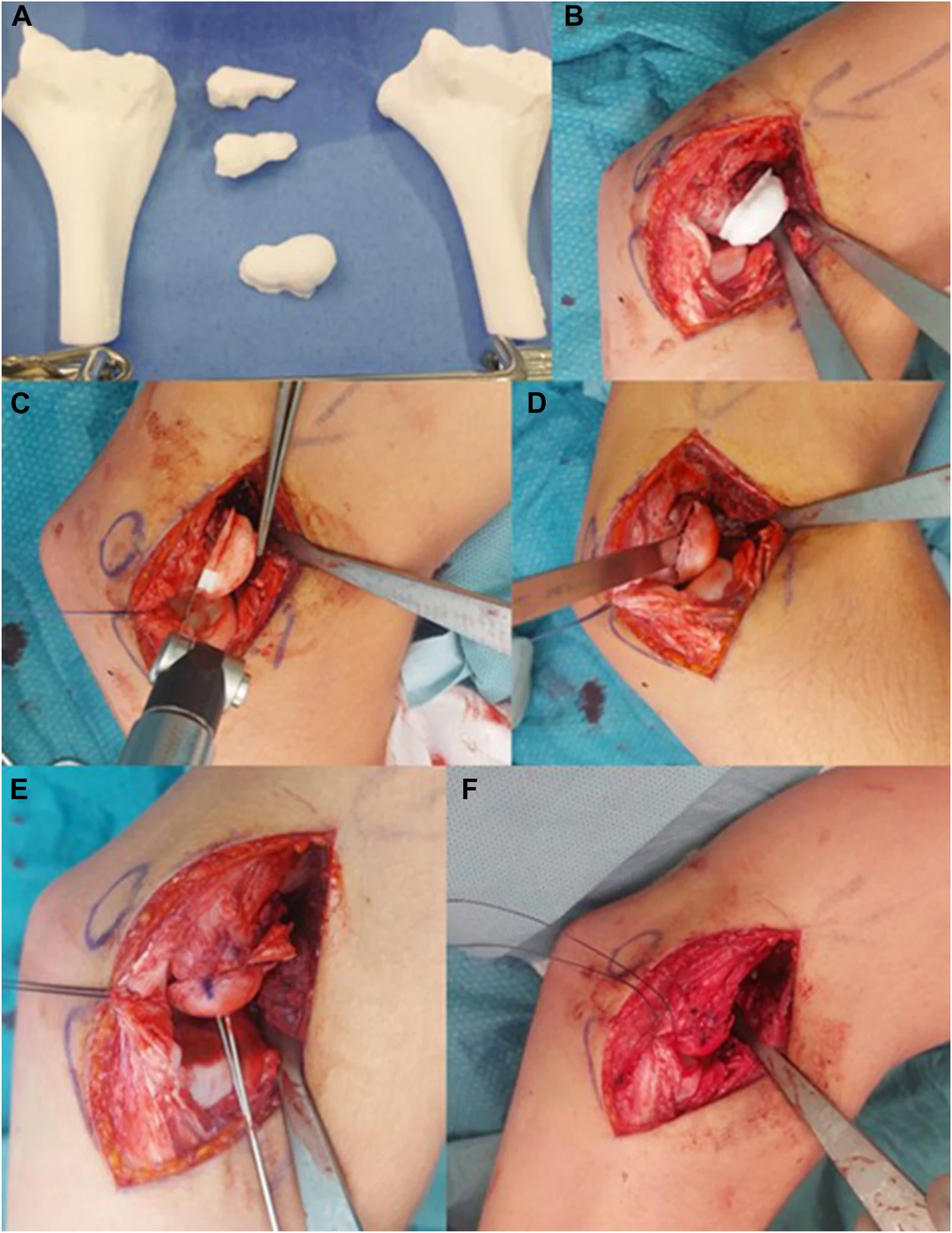
Perioperative imaging demonstrating the capitellar reconstruction, performed via the Kaplan approach with takedown of the LCL. (**A**) 3D-printed molds of the malunited capitellum as a guide for the osteotomy. (**B**) Placement of the 3D reconstructed mold on the malunited capitellar fragment. (**C**) Osteotomy following the surgical 3D-printed mold. (**D**) Capitellar fragment after osteotomy has been performed. (**E**) Reduction of the capitellar fragment on its anatomic site. (**F**) Fixation of the capitellum on its anatomic site using 2 cannulated compression screws and refixation of the LCL. *3D*, three-dimensional; *LCL*, lateral collateral ligament.

**Figure 10:**
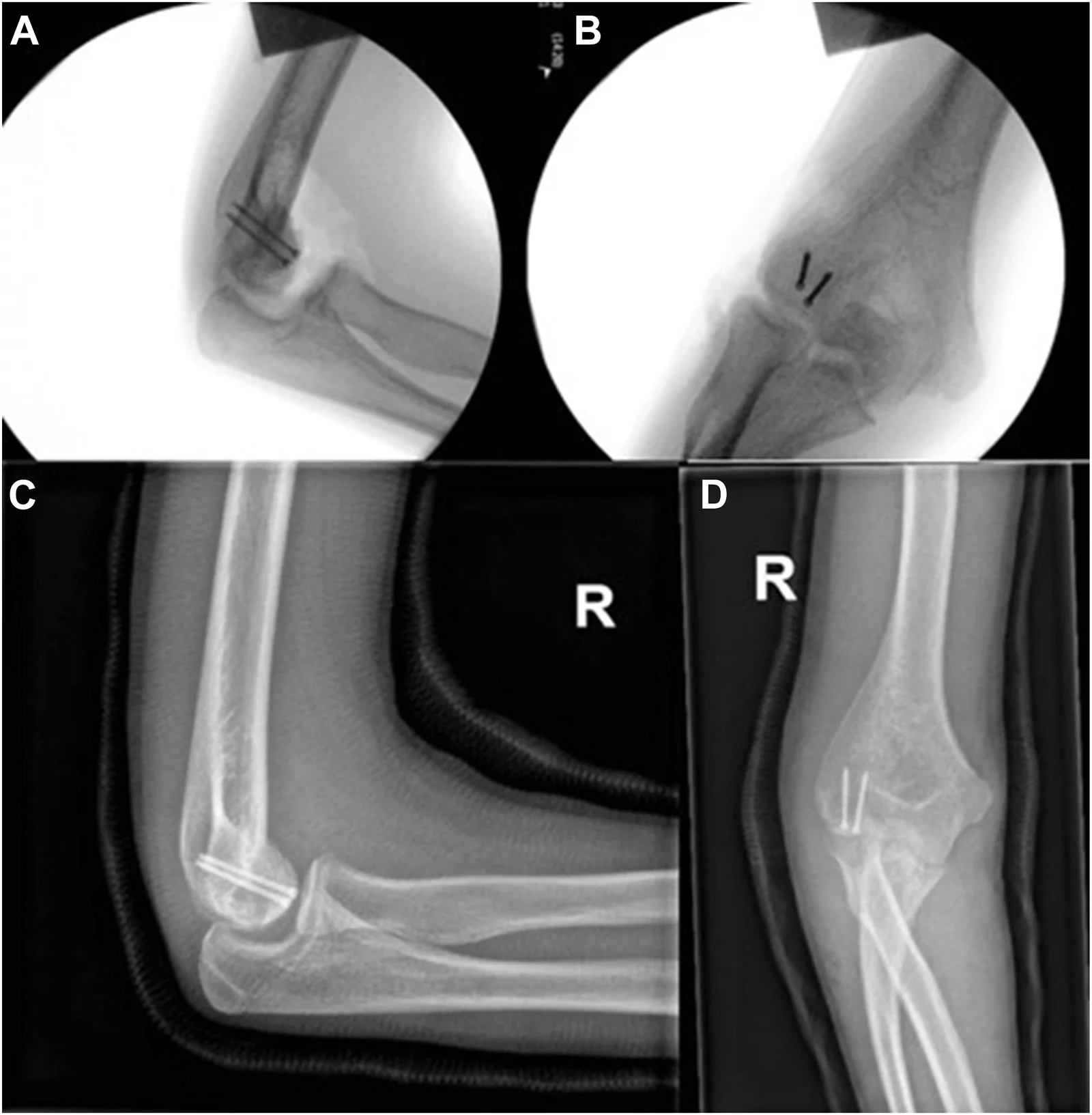
(**A** and **B**) Intraoperative lateral and anteroposterior (AP) imaging of the elbow joint after fixation with cannulated compression screws. (**C** and **D**) Lateral and anteroposterior (AP) imaging at 3-month follow-up.

Pre-operative planning was conducted using 3D reconstructions and patient-specific 3D-printed guides (Fig. 8, *A*-*F*), following the same technical protocol as case 1. The surgical procedure (Fig. 9, *A*-*F*) was performed via a Kaplan approach. Initial arthrolysis and capsulotomy were carried out, which successfully restored intraoperative flexion to 140° and fully resolved the extension deficit. Using the patient-specific guides, the malunited capitellum was osteotomized and anatomically reduced to its native site. Fixation was achieved with 2 cannulated compression screws, and the procedure was completed with the anatomic refixation of the lateral (ulnar) colateral ligament (L(U)CL) using a suture anchor. Post-operative testing of the collateral ligaments showed a stable elbow-joint. Intraoperative imaging can be seen in Figure 10, *A* and *B*.

Postoperatively, 2 weeks of long arm casting in 90° of flexion was followed by a 4-week period of dynamic splinting combined with overhead mobilization exercises. In contrast to case 1, this patient achieved a more favorable functional outcome with only a mild residual extension deficit, likely due to the significantly shorter delay between the initial trauma and the definitive surgical reconstruction.

The 6-week follow-up showed no pain complaints, with flexion of 110°, an extension deficit of 60°, pronation of 80°, and supination of 85°. Progressive static bracing therapy was started. The radiographs at the 3-month follow-up showed a complete consolidation of the osteotomy (Fig. 10, *C* and *D*). Patient satisfaction was high with no pain reported. Flexion improved to 120° without an extension deficit, and pronation and supination were completely restored to 90°. At 1-year follow-up, satisfaction was again high, with no reported pain. Flexion of 130° was found with an extension deficit of 20°. No deficits in pronation and supinations were found.

### Patient-related outcome measures

**Figure 11:**
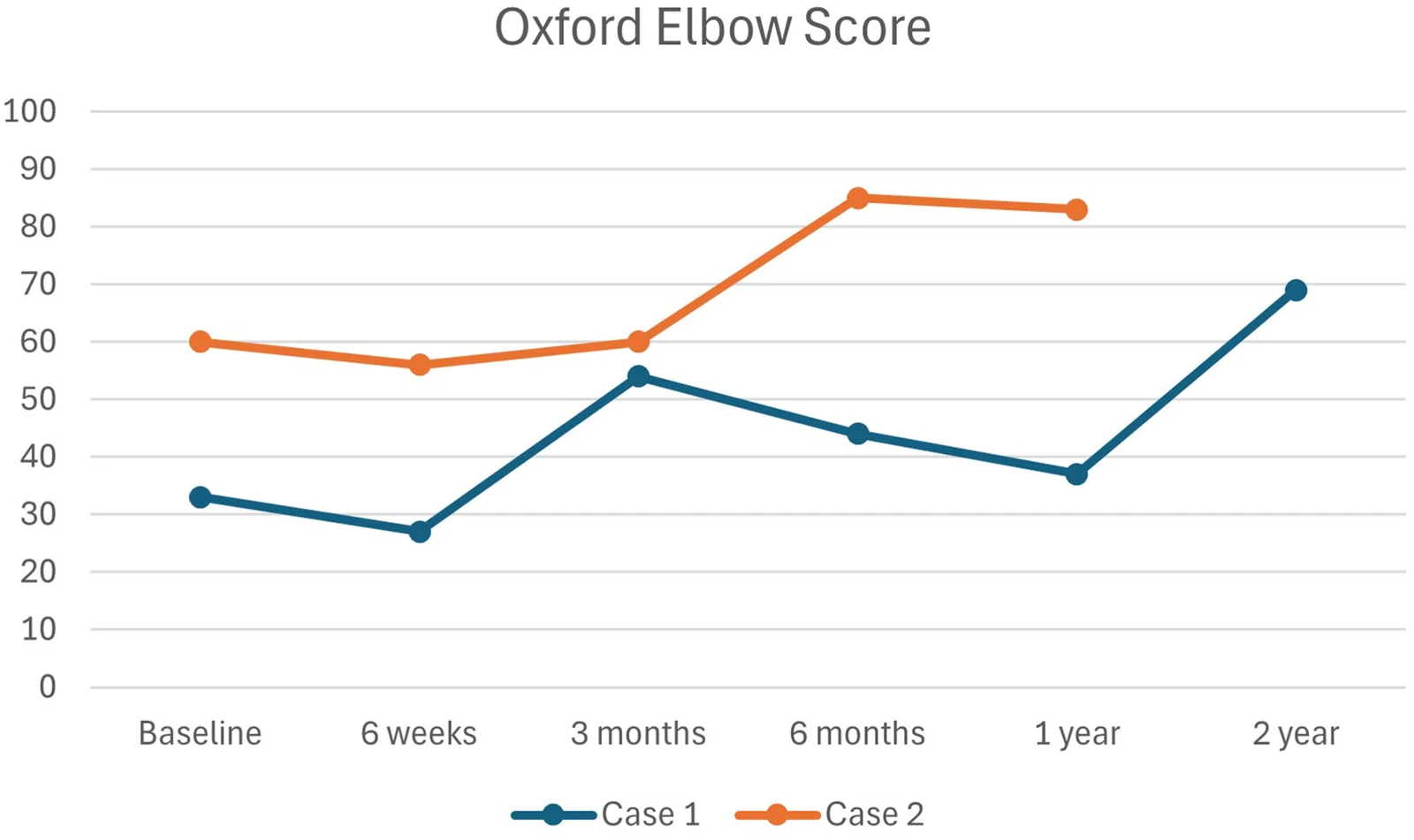
The Oxford Elbow Score (OES) displaying the scores given at different time stamps, with both cases displaying increased scores.

**Figure 12:**
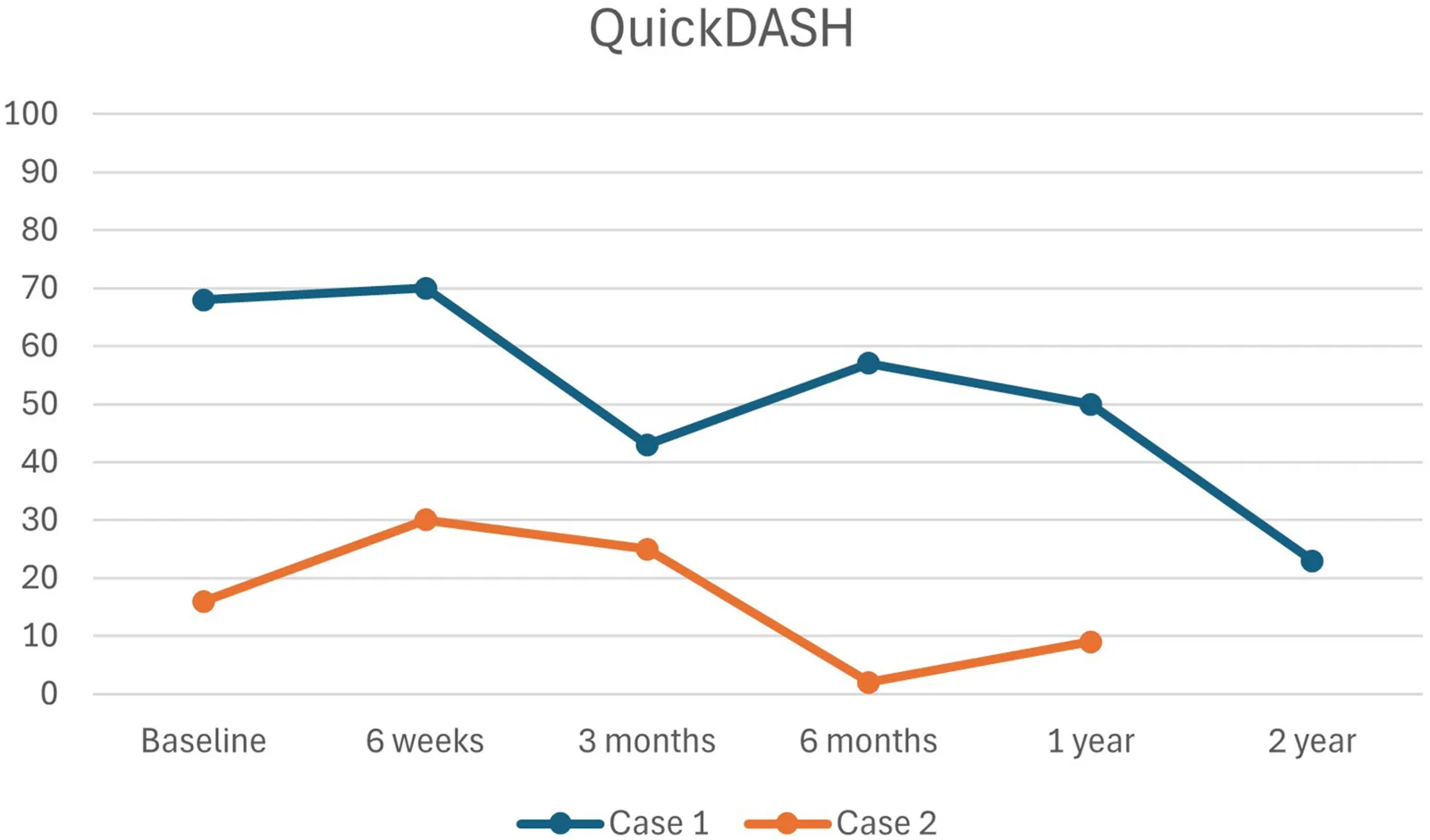
The QuickDASH displaying the scores given at different time stamps, with both cases displaying decreased scores, indicating less disability. *QuickDASH*, Quick Disabilities of the Arm, Shoulder and Hand.

**Figure 13:**
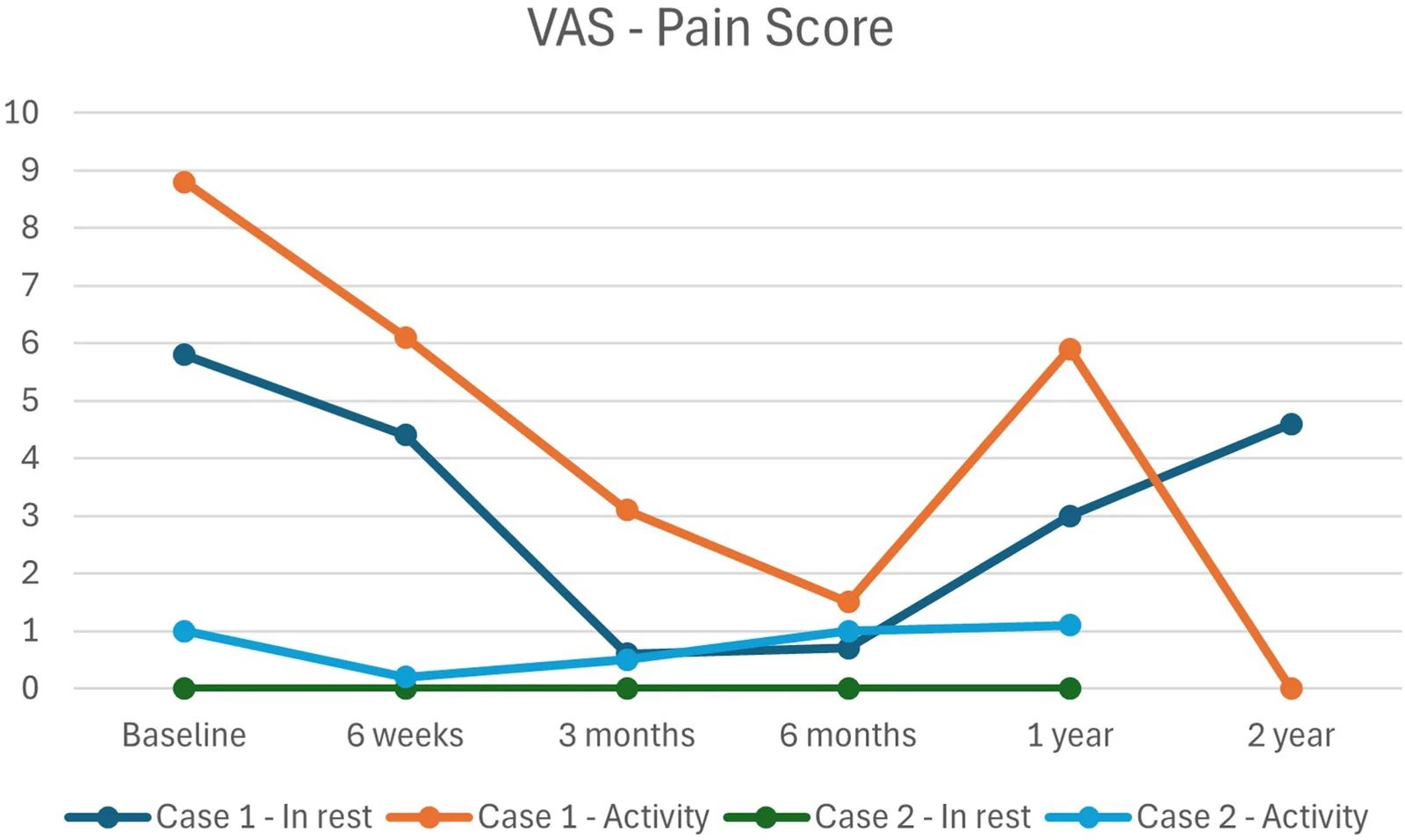
Visual analog scale (VAS) pain score ranging from 0 to 100. The reported pain scores in rest and during activity are displayed for both cases.

**Figure 14:**
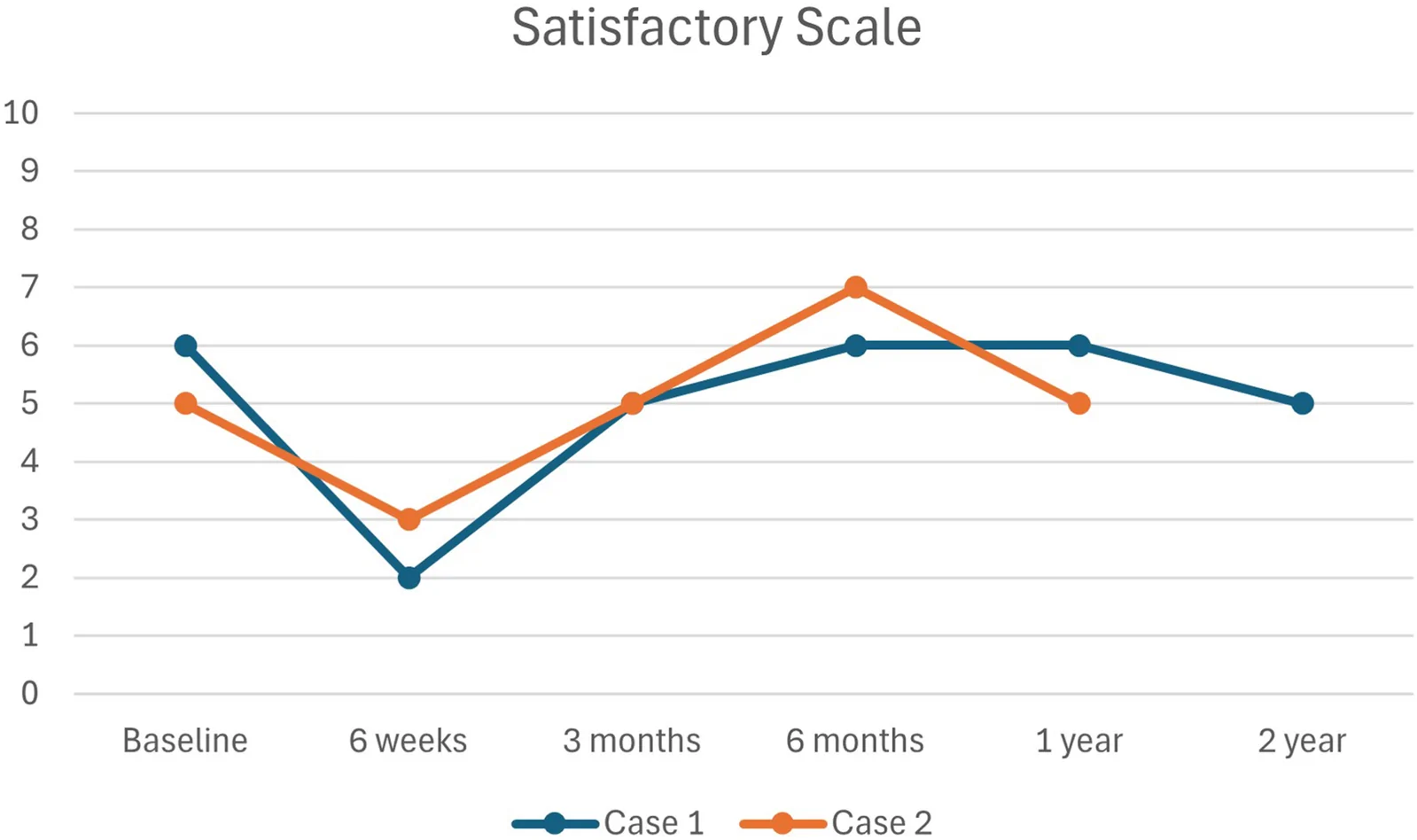
The satisfactory scale ranging from 0 to 10 for both cases.

PROMs of both cases are shown in Figure 11, Figure 12, Figure 13, Figure 14.

## Discussion

This report describes 2 adolescent patients with delayed presentations of malunited capitellum fractures; each treated with 3D patient-specific–guided corrective osteotomy and reconstruction. The contralateral CT scan was used as a template and overlaid both images for comparison and surgical planning. The image registration and overlay were performed using Mimics software (Materialise NV, Leuven, Belgium). The planning itself was carried out in our in-house 3D lab. No external company was involved in the planning process. The patient-specific guides were designed in such a way that the first guide precisely encompassed the malunited capitellum. By performing the osteotomy along the edge of this guide, the segment to be repositioned could be accurately separated from the distal humerus. The second guide indicated which portion of post-traumatically formed callus distal to the malunion needed to be removed to enable anatomic reduction. For additional visualization and pre-operative planning, 3D prints of both the pre-operative anatomy and the planned post-operative anatomy were created.

These 2 cases underscore the utility of 3D pre-operative planning and patient-specific guides in the management of pediatric elbow malunions. They also illustrate the variability in outcomes related to timing of diagnosis and severity of intra-articular changes.

These findings are consistent with existing literature on intra-articular malunions of the elbow, in which restoration of anatomic alignment is considered essential for optimal functional recovery.^15^^,^^17^ Previous studies have demonstrated that anatomic reduction of capitellar fractures is strongly associated with improved range of motion and reduced pain; however, outcomes remain dependent on the timing of intervention and the extent of secondary joint degeneration.^6^^,^^11^^,^^12^ In recent years, 3D pre-operative planning and patient-specific instrumentation have gained increasing attention in orthopedic trauma surgery, particularly in the management of complex, multiplanar deformities. These techniques allow for improved visualization of the deformity, simulation of the osteotomy, and more accurate execution of the planned correction. Several studies have reported reductions in operative time and improved surgical precision when 3D-assisted techniques are applied, although evidence in pediatric elbow pathology remains limited.^3^^,^^8^^,^^10^

Despite correction of the bony alignment, among the surgeries presented, case 1 showed persistent stiffness in early follow-up. The prolonged delay of eight months between injury and surgery likely resulted in irreversible capsular fibrosis, intra-articular adhesions, and early degenerative joint changes. These factors explain the persistent stiffness despite achieving an anatomic reduction. Onay et al^11^ found that adolescent patients treated operatively for capitellum fractures showed good-to-excellent outcomes at midterm and long-term follow-up, provided the reduction was anatomic and early rehabilitation was started. It highlights the need for timely intervention and reduction of bony alignment, as tissue changes may become irreversible even with perfect bony alignment.

Post-operative PROMS showed satisfactory functional improvement, more so in case 2, in which the sole complaint was a mild extension deficit. The short-term follow-up highlights the importance of early intervention, as the functional results in case 2 were better than in case 1. Longer follow-up is needed to assess longevity of the reconstructions and degenerative changes over time. Interestingly, patient satisfaction scores at 1 year returned to baseline levels, despite objective improvements in pain and range of motion. This finding likely reflects the multifactorial nature of patient-reported outcomes in this population. In particular, the delayed presentation in case 1 resulted in established stiffness, capsular contracture, and early degenerative joint changes, which are only partially reversible and may limit perceived recovery. Furthermore, given the young age and high functional demands of these patients, modest improvements may not translate into a subjective sense of full recovery. This is supported by the observation that other PROMs, such as the OES and QuickDASH, demonstrated improvement over time, suggesting functional benefit despite stable satisfaction scores. These findings highlight the importance of considering patient expectations and baseline pathology when interpreting subjective outcome measures.

### Recommendations and future directions

These cases suggest that 3D technologies offer promising adjuncts to the surgical management of complex or delayed capitellar injuries in adolescent patients. However, further studies are needed to evaluate long-term functional and radiographic outcomes, standardize protocols for 3D-guided surgical planning, and investigate cost-effectiveness and learning curves.

Given the rarity of these injuries, multicenter collaborations or registries may be necessary to generate statistically robust data.

## Conclusion

Malunited capitellum fractures in the adolescent population are severe elbow pathologies with a challenging treatment. 3D-guided osteotomy and reconstruction seem to be a contributing surgical technique in early follow-up. Further follow-up and additional cases are needed to gain more insight into the effects of this technique.

## Disclaimers:

Funding: No funding was disclosed by the authors.

Conflicts of interest: The author, their immediate family, and any research foundation with which they are affiliated have not received any financial payments or other benefits from any commercial entity related to the subject of this article.

Patient consent: Obtained.

## Declaration of Generative AI and AI-Assisted Technologies in the Writing Process

During the preparation of this work the author(s) used Grammarly for grammatical correction After using this tool/service, the author(s) reviewed and edited the content as needed and take(s) full responsibility for the content of the publication.
